# Psychiatric care in Croatia during COVID-19 lockdown and earthquake: significant decrease in admissions to University Psychiatric Hospital Vrapce in Zagreb

**DOI:** 10.1192/j.eurpsy.2022.508

**Published:** 2022-09-01

**Authors:** D. Polšek, A. Botica, T. Sabo, M. Baković, V. Marinović, G. Arbanas

**Affiliations:** 1 University Psychiatric Hospital Vrapče, Department For Psychotic Disorders, Zagreb, Croatia; 2 University Hospital Centre Split, Dpt. Of Psychiatry, Split, Croatia; 3 Psychiatric Hospital Ugljan, Dpt. Of Psychiatry, Ugljan, Croatia; 4 University of Rijeka, Medical Department, Rijeka, Croatia; 5 University Psychiatric Hospital Vrapče, Department For Forensic Psychiatry, Zagreb, Croatia

**Keywords:** COVID- 19 pandemic, Emergency psychiatric care, Hospital admission, lockdown

## Abstract

**Introduction:**

The COVID-19 pandemic has had an enormous impact on both physical and mental health of people around the world.

**Objectives:**

The aim of the study was to evaluate the number and characteristics of people seeking emergency psychiatric help during combined psychosocial stressful events in March 2020.

**Methods:**

Data for 3927 patients seeking emergency psychiatric help were collected and analyzed for the months preceding, during and after lockdown due to COVID-19 pandemic and concomitant earthquake that took place on 22nd March 2020 in Zagreb, and compared with the same months of 2019.

**Results:**

A significant decrease in both the number of visits and admissions to the hospital was found for the month of lockdown. There was a significant decrease in the number of out-patients visits and day hospital admissions. Compared with other months, more women and younger patients sought help. There was a significant rise in the number of patients presenting with suicidal thoughts, as well as a larger percentage of involuntary admissions.

**Conclusions:**

Overall less people sought psychiatric help in the face of an unpredictable acute threat, which was interpreted in the light of prioritizing fear of infection over mental health issues. Alternatively, it is possible that people threatened with immediate danger mobilize short- term compensatory psychological resources which help deal with or put off mental illness. This research was conducted as part of the project of the Croatian Science Foundation CORONA-04-2086 Life in the time of COVID-19-social implications on the security and well-being of vulnerable groups in the European context.

**Disclosure:**

No significant relationships.

